# Target Analysis and Mechanism of Podophyllotoxin in the Treatment of Triple-Negative Breast Cancer

**DOI:** 10.3389/fphar.2020.01211

**Published:** 2020-08-07

**Authors:** Wenfeng Zhang, Cun Liu, Jie Li, Ruijuan Liu, Jing Zhuang, Fubin Feng, Yan Yao, Changgang Sun

**Affiliations:** ^1^Clinical Medical Colleges, Weifang Medical University, Weifang, China; ^2^College of First Clinical Medicine, Shandong University of Traditional Chinese Medicine, Jinan, China; ^3^College of Traditional Chinese Medicine, Shandong University of Traditional Chinese Medicine, Jinan, China; ^4^Department of Oncology, Weifang Traditional Chinese Medicine Hospital, Weifang, China; ^5^Chinese Medicine Innovation Institute, Shandong University of Traditional Chinese Medicine, Jinan, China

**Keywords:** breast cancer, cell cycle, inhibition, network pharmacology, podophyllotoxin

## Abstract

**Background:**

As the original compound of many podophyllotoxin derivatives, podophyllotoxin has a beneficial antitumor effect. The mechanism of podophyllotoxin activity in triple-negative breast cancer still needs to be explored.

**Methods:**

We used cell proliferation assay, scratch and transwell experiments, and cell cycle and apoptosis analyses to observe the intervention effect of podophyllotoxin on breast cancer. Furthermore, we analyzed the differences between GSE31448, GSE65194, and GSE45827 in the Gene Expression Omnibus database (GEO) and explored the differential genes using a STRING database. Centiscape2.2, MCODE cluster analysis and KEGG pathway analysis were used to identify the most significant gene differences. Next, we utilized BATMAN-TCM and TCMSP databases for further screening to identify key genes. Finally, quantitative RT-PCR (qRT-PCR) and Western blotting were performed to detect the expression of key targets.

**Results:**

Our research confirmed that podophyllotoxin could not only inhibit the migration and invasion of triple-negative breast cancer but also affect the cell cycle and induce apoptosis. In total, 566 differential genes were obtained by using the GEO database. After topological network analysis, cluster analysis, and molecular docking screening, we finally identified PLK1, CCDC20, and CDK1 as key target genes. The results of the qRT-PCR assay showed that the mRNA levels of PLK1, CDC20, and CDK1 decreased, while the expression of upstream P53 increased, after drug induction. The Gene Set Enrichment Analysis (GSEA) and conetwork analysis showed that PLK1 is a more critical regulatory factor. Further Western blotting analysis revealed that there was a negative regulatory relationship between the key gene PLK1 and P53 on the protein level. The results were presented as the mean ± standard deviation of triplicate experiments and P<0.05 was considered to indicate a statistically significant difference.

**Conclusion:**

Podophyllotoxin has an intervention effect on the development of triple-negative breast cancer. The expression of PLK1, CDC20, and CDK1 in the cell cycle pathway is inhibited by regulating P53. Our research shows that natural drugs inhibit tumor activity by regulating the expression of cyclins, and the combination of natural drugs and modern extensive database analysis has a wide range of potential applications in the development of antitumor therapies.

## Introduction

Breast cancer is a malignant tumor that seriously threatens women’s health around the world. Although in the past decades, the diagnosis and treatment of breast cancer have been substantially improved, the median survival time of triple-negative breast cancer (TNBC) is less than one year (Kennecke et al., 2010). TNBC is a type of breast cancer with negative estrogen receptor (ER), progesterone receptor (PR), and human epidermal growth factor receptor 2 (HER2) genes (Brown et al., 2008). All the three key therapeutic targets are negative and have highly invasive clinical characteristics. At present, targeted and hormone types of therapy have no beneficial therapeutic effect on TNBC. Interestingly, TNBC may be sensitive to drugs related to DNA damage and mitotic spindle toxicity (De Laurentiis et al., 2010). More attention has been paid to some natural plant drugs that can interfere with critical molecules in cell growth and development signaling pathways. Natural plant drugs are widely used in inhibiting tumor activity (Muniraj et al., 2019). Podophyllotoxin is a kind of natural aryl lignin, which is the leading compound of many derivatives (Li et al., 2013). Botanic compounds are important components of drug development (Antunez-Mojica et al., 2016). Podophyllotoxin inhibits microtubule polymerization, leading to mitotic failure and cell cycle arrest (Chen et al., 2013). Related drugs can be used as new targets and approaches for future TNBC research.

In addition, genomics and big data are already advancing our understanding of many diseases and escalating the development of new drugs for TNBC. Gene expression profile analysis using microarray technology provides a new and promising field for cancer research. Improving and expanding access to this information through digital technologies leads to increasingly personalized therapies that are tailored to an individual unique genetic profile (Nannini et al., 2009). A large number of datasets are stored in public databases, such as tumor genome map, gene expression comprehensive database (GEO) or ArrayExpress. Applying this comprehensive analysis can better grasp the mechanisms of tumorigenesis and help to identify new drug targets, molecular diagnosis, and prognosis in limited tumor samples.

In our study, in order to study the effect of natural small molecule compounds on triple negative breast cancer and explore key targets of podophyllotoxin intervention in TNBC, we first used podophyllotoxin to interfere with TNBC cell lines. It was found that podophyllotoxin could significantly inhibit the migration and invasion of TNBC cells, block cell cycle, and induce apoptosis. In order to further explore its underlying mechanism of action, we used UALCAN to predict the expression and prognosis of key genes through the construction of drug-disease-target relationship network. The potential targets of podophyllotoxin activity on TNBC were identified. Later, it was verified by qRT-PCR analysis that CDK1, PLK1, and CDC20 were the direct targets of podophyllotoxin in breast cancer cells. The innovation of microarray technology and the construction of public repository of microarray data, as well as the network construction and analysis of multiobjective components and key targets provide better understanding of the mechanisms of potential disease development (Liu et al., 2019). Our research has constructed a “drug-targeting-disease” network, which has opened up ideas for the study of small natural molecules. The effective combination of pharmacology and bioinformatics provides a feasible new idea for exploring the pathogenesis of tumors in the future and generates a scientific basis for the exploration of new tumor targets.

## Methods

### Cell Lines

The TNBC cell lines, MDA-MB-231 and MDA-MB-468, were sourced from the American Type Culture Collection (ATCC, United States). The cells were maintained in RMIO medium (Gibco, USA) containing fetal bovine serum (SIJIQING, China) and penicillin/streptomycin to the final contents of 10% and 1%, respectively. The cells were incubated under standard conditions (5% CO2, 37°C, 95% humidity). In all experiments, the cells were in the growth stage.

### Cell Viability Assay

The effect of podophyllotoxin(purity:≥ 98%, Solarbio, China) on cell viability was determined by 3‐(4,5‐dimethylthiazol‐2‐yl)‐2,5‐diphenyltetrazolium bromide assay (Sigma‐Aldrich), for which MDA MB-231 and MDA-MB-468 cells were cultured in 96-well plates. The density of MDA-MB-231 cells was 1.2 × 10^4^ per well, while that of MDA-MB-468 cells was 1.5 × 10^4^ cells per well. Before adding the drug, the cells were cultured in a serum-free medium for 6h and treated with varying concentrations of podophyllotoxin (0.75, 1.5, 3, 6, 12, and 24 μmol/ml) for 48 h. After that, each well was incubated with 15 μl of MTT for 4 h, and 150-μl dimethyl sulfoxide was added after incubation. After shaking, the microplate reader was used to detect the absorbance at 570 nm. The calculation formula is as follows:

$$\frac{\text{(average OD value of control group) - (average OD value of experimental group)}}{\text{(average OD value of control group) - (average OD value of blank control group)}}\times 100\%$$

The above OD values are average OD values. The result is plotted by Graphpad prism.

### Migration Experiment

At the 90% confluency, the wound was scratched with the pipette tip in the MDA-MB-231 and MDA-MB-468 cell cultures, which were subsequently treated with 0 μmol/ml or 0.75 μmol/ml for 24 h. Finally, the migration distance from the perimeter to the middle was measured by using imaging software (ImageJ; National Institutes of Health, Bethesda, MD, USA).

### Invasion Experiment

We used MDA-MB-231 and MDA-MB-468 cell lines to observe the invasive ability of tumor cells. Before the experiment, the cells starved for 12 h in the serum-free medium. After the application of the substrate glue (BD Biosciences, Franklin Lakes, NJ, USA), the cell suspension was added in the upper chamber and the serum-free medium was added in the lower chamber of the device (Costar). The cells were incubated at 37°C for 24 h. After incubation, the cells were stained with crystal violet to observe the invasive ability of the cells.

### Apoptosis and Cell Cycle Experiments

Apoptosis and cell cycle were detected by Annexin V FITC APOP DTEC kit (BD, 556547) and cell cycle staining kit (BestBio, BB-4104-3). Upon reaching 80%–90% of confluency in a six-well plate, the cells in the control and drug groups were collected, respectively. The dye was added and incubated for 5 min, and the apoptosis distribution and cycle stages were detected by the flow cytometry detector.

### Dataset and Variance Analysis

The GEO data platform is a complementary resource for storing and retrieving data from high-throughput gene expression and genomic hybridization data (Edgar et al., 2002). GEO database contains many high-throughput gene chips uploaded after peer-reviewed studies. We manually queried the GEO database (www.ncbi.nlm.nih.gov/geo/) for the keywords - “Triple negative breast cancer mRNA” and used the following criteria to filter the datasets: (a) the study used human samples; (b) data were from TNBC tissues and non-breast cancer tissues. We selected three target gene expression datasets (GSE31448, GSE65194, and GSE45827) involving 186 sample chips, including 26 healthy breast tissue samples and 164 TNBC tissue samples from the GEO platform. GEO2R, an interactive web tool, is used to analyze GEO data. We define grouping according to sample type. All the genes of this chip need to be preserved, and our chip is standardized and logarithmically converted. We screen the differential genes of each chip according to the criteria of p<0.05 and logFC>2 or logFC<-2. The differences of GSE 45827, GSE 65194, and GSE 31448 were analyzed respectively. The final differential genes were obtained by using intersection in Venn diagram.

### Protein-Protein Interaction Network

Module analysis of the STRING database (http://www.stringdb.org/) realizes the functional correlation between proteins on a global scale (Szklarczyk et al., 2015). The STRING database integrates a large number of protein-protein correlation data based on coexpression analysis and cross-genomic evolutionary signals. (Doncheva et al., 2019)Besides, this database is also helpful to analyze the modularity in biological processes.(von Mering et al., 2003) In protein-protein interaction (PPI) networks, the comprehensive score over 0.4 was considered statistically significant. Then, the differential genes were visualized by Cytoscape software. Cytoscape is a convenient tool that integrates biological network visualization and data integration. The design of custom node graphics provides more possibilities for new network visualization(Smoot et al., 2011). Then the intermediate betweenness UnDir, degree UnDir, and other items were scored, resulting in the top ten items with the highest score as the intersection point. We also used the MCODE(Bader and Hogue, 2003) plug-in in Cytoscape to screen important gene modules. MCODE score > 5, degree cut-off=2, node score cut-off = 0.2, and maximum depth=100, and k-score=2 were selected as criteria to produce important modules. We carried out KEGG and GO analyses to determine the biological process with significant enrichment of module genes.

### GO and KEGG Enrichment Analysis of DEGs

We used the David database (https://david.ncifcrf.gov/), FUNRICH (http://www.funrich.org/) and Metascape (http://metascape.org/) for GO and for KEGG analyses. David database is a web-based online bioinformatics resource, which integrates bioinformatics information and online tools, providing significant supporting information integration (Jiao et al., 2012). In particular, the David database analysis module obtains valuable data from the specified information at different scales, integrating the focus and intensity between different modules (Huang da et al., 2009). GO database is introduced in detail from three aspects: cell function, participation in the biological pathway, and cell localization. In parallel, we have a more intuitive and comprehensive understanding of the metabolic pathways involved in this pathway, which is helpful in obtaining more comprehensive biological information.

In addition, UALCAN is also used by us to analyze the expression of key genes. UALCAN (http://ualcan.path.uab.edu/index.html) is an effective website for online analysis and mining of cancer data. UALCAN is based on PERL-CGI, JavaScript and CSS, using the TCGA database to analyze relevant cancer data, UALCAN studies the gene expression of any specific cancer and its disease relevance. (Chandrashekar et al., 2017)After we select the corresponding TNBC samples, we will obtain the expression of related genes.

### Molecular Docking and Comprehensive Analysis of GSEA Enrichment

We used molecular docking software for molecular docking based on molecular dynamics. Molecular docking is a more accurate method to understand the reaction mechanism of proteins or enzymes with ligands. Because of its easy use and low cost, it has been widely used in many fields (Liu et al., 2018). It can also predict protein binding mode and affinity. We used Tripos’ Sybyl-x2.0 software for molecular modeling, the two-dimensional structure of podophyllotoxin compounds was downloaded directly from the PubChem database, and the target crystal structure was obtained from RCSB protein database (https://www.rcsb.org/). In the process of molecular model construction, PLK1 (PDB:4o6w), CDK1 (PDB:6guh), CDC20 (PDB:4r8q) were used. Next, the Surflex-Dock docking mode was adopted, and finally, the Surflex-Dock score (total score) was set as standard. After obtaining the docking molecular protein structure, we used PYMOL software for its further optimization.

Then we used the online traditional Chinese medicine analysis software BATMAN-TCM (http://bionet.ncpsb.org/batman-tcm) and TCMSP database to predict and analyze podophyllotoxin targets. Batman, as an analytical tool, is able to integrate the relevant information of known prescriptions for diseases but also helps to understand the overall action mechanism of traditional Chinese medicine or prescriptions (Liu et al., 2016). In addition, we also carried out a GSEA enrichment analysis. GSEA is a flexible calculation method, which is used to determine whether a group of predefined genes has statistical significance and consistency between the two biological states, which makes up for the deficiency of traditional gene expression analysis(Subramanian et al., 2005). Whereas GO and KEGG analyses may miss some genes with insignificant but significant differences, GSEA comprehensively demonstrates the overall trend of genes so that researchers can better grasp the function and significance of gene network. P-value < 0.05 and FDR < 0.25 were regarded as the reliable standard.

### mRNA Expression Analysis

After MDA-MB-231 and MDA-MB-468 were treated with podophyllotoxin for 24 h, the total mRNA was extracted and separated by the Trizol kit. The total mRNA was isolated, and the target genes were obtained by using PCR amplification with reverse transcriptase cDNA, and cDNA as a template. The quantitative (Q) primer sequence are as follows:

GRAPH: (forward: 5’AGAAGGCTGGGGCTCATTTG3’; reverse: 5’AGGGGCCATCCACAGTCTTC3’);PLK1: (Forward: 5’TTTGGGCAAGGGCGGCTTTG3’; reverse: 5’GCGGCTTGAGCAGCAGAGAC3’); CDC20: (Forward: 5’AGCAGCAGATGAGACCCTGAGG3’; reverse: 5’CAGCGGATGCCTTGGTGGATG); CDK1: (Forward: 5’GTGCTTATGCAGGATTCCAGGT3’; reverse: 5’CCATGTACTGACCAGGAGGGA3’); P53: (Forward: 5’TGCGTGTTTGTGCCTGTCCTG3’; reverse: 5’TTGTTGGGCAGTGCTCGCTTAG3’) after amplification, it is detected by PCR instrument. The Ct value of the internal reference gene is used to normalize the target gene Ct value of the experimental group (test) and the control group (con). Then ΔΔ Ct=Δ Ct test-Δ Ct con. Finally, calculate the difference in expression levels, Change Fold = 2^-ΔΔCt^.

### Western Blotting

The cells were lysed with RIPA buffer, the supernatant was collected, centrifuged by 12,000 rpm for 15 min, and SDS-PAGE was performed. After that, the proteins were transferred to the PVDF membrane and blocked with milk powder (5% skimmed milk powder, 1.5 h). The first antibodies of PLK1 and P53 were incubated according to the dilution ratio of 1:2000, for 4°C overnight (β-actin, PLK1, and P53 were all purchased from Abcam), and then incubated with corresponding secondary antibodies for 1 h.

### Statistical Analysis

For continuous data obtained from *in vitro* experiments, significant differences were determined using the Student’s t-test. GraphPad Prism 6.0 statistical software (GraphPad Software, La Jolla, California) and ImageJ software (National Institutes of Health) were used for quantitative analysis of the data. All experiments in this research were independently repeated at least three times. We consider statistical difference at P < 0.05.

## Results

### Podophyllotoxin Inhibits the Proliferation, Migration, and Invasion of TNBC Cells

**Figure 1:**
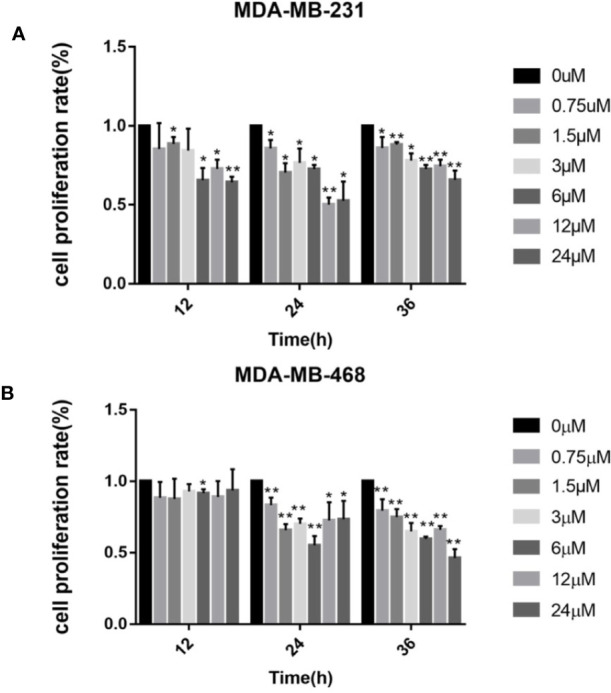
Cell viability assay (MTT) of **(A)** MDA‐MB‐231 and **(B)** MDA‐MB‐468 cells treated with 0–24 μM of podophyllotoxin 12–36 h. The inhibition rate of the two kinds of cells treated by drugs was the highest at 24 h. Each data represents the mean ± SD from three independent experiments *p < 0.05, **p < 0.01.

**Figure 2:**
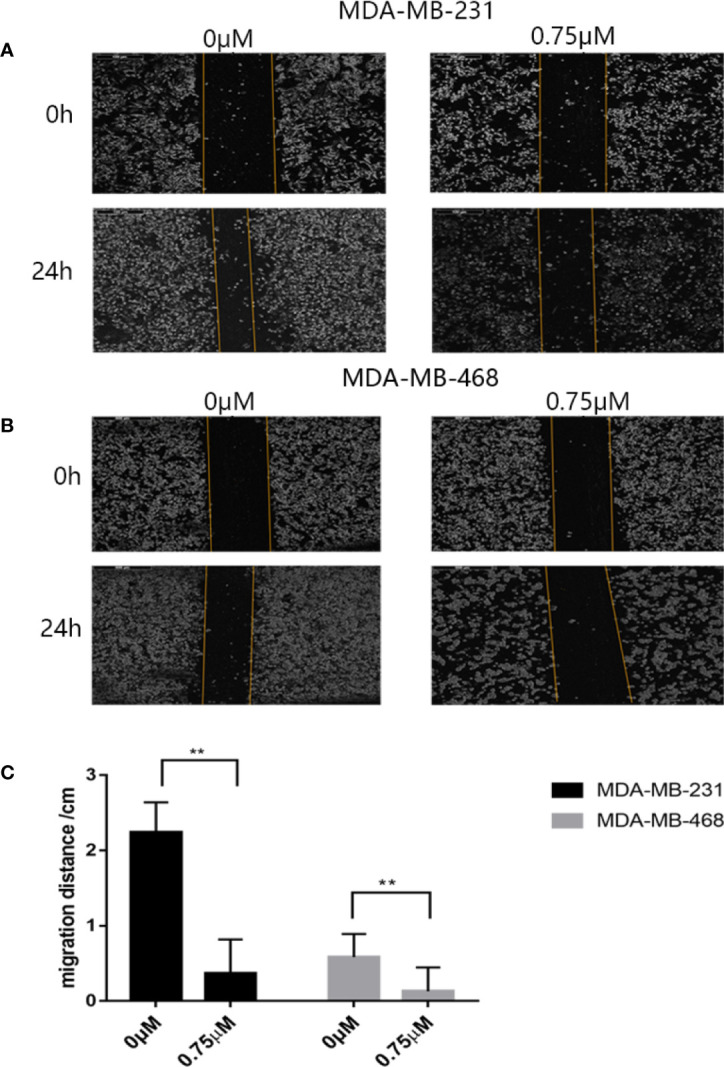
Antimigration effect of podophyllotoxin. After 24 h of drug intervention, the migration distance of the **(A)** MDA‐MB‐231 and **(B)** MDA‐MB‐468 in the control group was significantly narrower than that in the treatment group. Podophyllotoxin intervention could significantly inhibit tumor migration. The moving distance was measured by inverted microscope and photographed. **(C)** The proportion of migration between the cells treated with podophyllotoxin and the control group. Each data represents the mean ± SD from three independent experiments. **p < 0.01 was considered to indicate a statistically significant difference.

**Figure 3:**
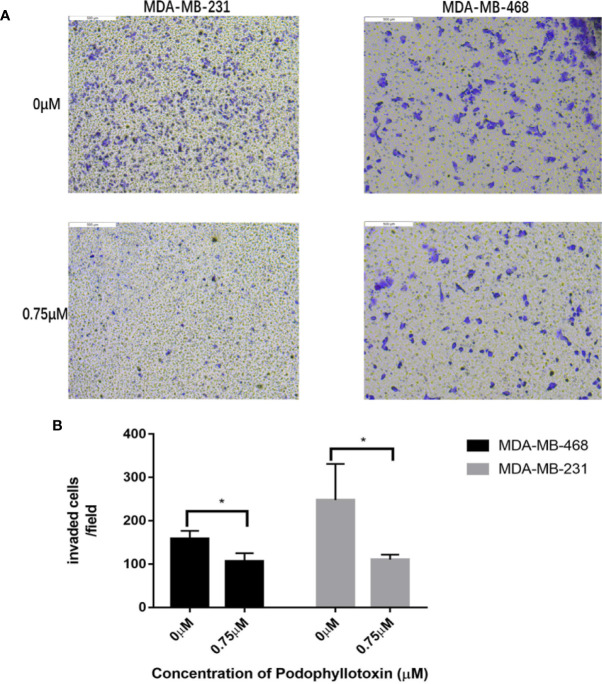
Antiinvasive effect of podophyllotoxin. *In vitro* invasion assay was used to observe the invasion of drugs on triple negative breast cancer cells (MDA-MB-231, MDA-MB-468). **(A)** After 24 h of intervention with podophyllotoxin, we found that the number of cells passing through the chamber decreased significantly, and podophyllotoxin could inhibit the invasion of triple negative breast cancer. **(B)** Bar graphs show the quantitative expression of by the number of invasive cells in transwell experiment. *p < 0.05 is considered statistically significant.

First we investigated whether podophyllotoxin has an effect on breast cancer cell lines MDA-MB-231 and MDA-MB-468 were treated with 0 μmol/L, 0.375 μmol/L, 0.75 μmol/L, 1.5 μmol/L, 3 μmol/L, 6 μmol/L, 12 μmol/L, and 24 μmol/L podophyllotoxin for 12, 24, and 36 h, respectively. The proportion of living cells was studied using the MTT colorimetric method. The results showed that the inhibition rates of 0.375 μmol/L and 0.75 μmol/L were better at 24 h (**Figures 1A, B**). The wound healing experiment showed that the migration ability of MDA-MB-231 and MDA-MB-468 decreased after 24 h of podophyllotoxin intervention (**Figures 2A, B**). The invasion experiment further proved that podophyllotoxin could inhibit the invasion of TNBC cells (**Figures 3A–C**). (all p < 0.05).

### Podophyllotoxin Can Block the Cycle of TNBC Cells and Induce Apoptosis

**Figure 4:**
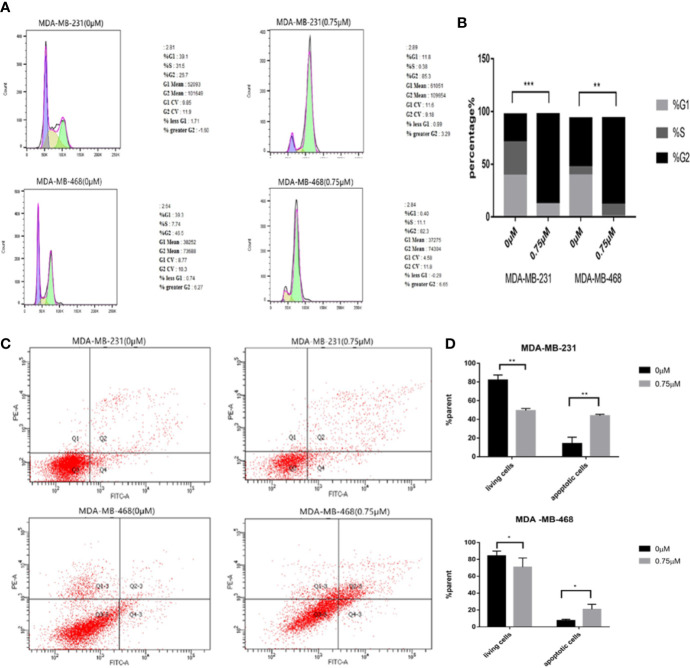
Effect of podophyllotoxin on the cycle and apoptosis of triple negative breast cancer cells. **(A)** After 0.75 μM podophyllotoxin treatment, the proportion of G1 phase decreased and the proportion of G2/M phase increased. Podophyllotoxin blocked triple-negative breast cancer cells in G2/M phase. **(B)** Statistics of cell cycle changes. P<0.05, the difference was statistically significant. **(C)** After 24 h of Podophyllotoxin (0 and 0.75 μM) intervention, and cell apoptosis was analyzed by flow cytometry. **(D)** Bar graphs show the quantification of apoptosis in MDA-MB-231and MDA-MB-468 cells. The data are expressed as the mean value ± SD.*p < 0.05, **p < 0.01, ***p < 0.001.

We need to further explore the effects of podophyllotoxin on cell cycle and apoptosis. When we treated with 0.75 μmol/L podophyllotoxin for 24 h, compared with the control group, we found that the cell cycle was blocked in the G2/M phase. As it is shown in **Figures 4A, B**, podophyllotoxin blocks the progress of the cell cycle. In the apoptosis experiment, we found that the number of apoptotic cells increased significantly after being treated with 0.75 μmol/L podophyllotoxin for 24 h. Thus, podophyllotoxin can induce apoptosis (**Figures 4C, D**). All values are presented as the mean ± standard deviation of at least triplicate samples and P<0.05 was considered to indicate a statistically significant difference, *p< 0.05, **p< 0.01, ***p< 0.001.

### Identification of DEGs

**Figure 5:**
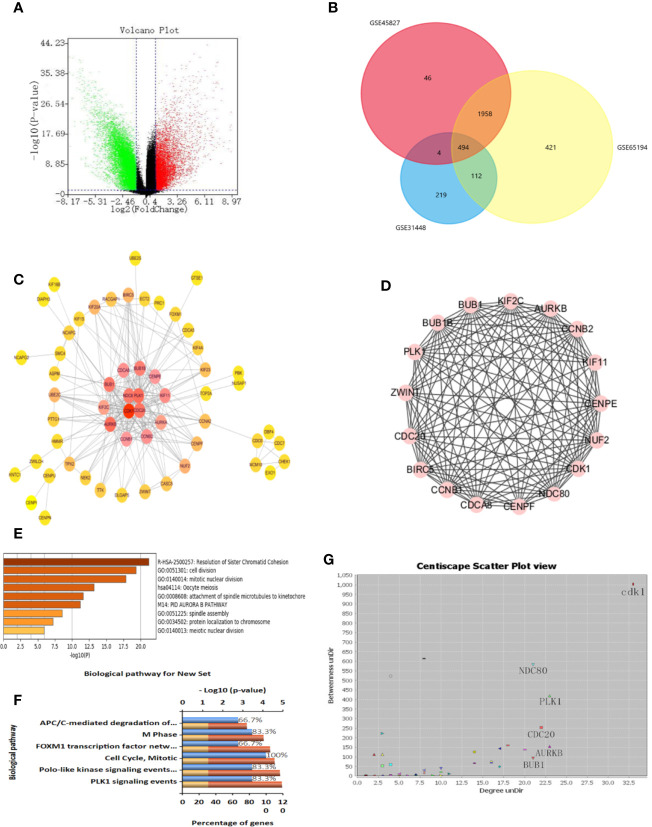
Identification of differential genes and key genes. **(A)** Volcanic maps of all the genes and identification of DEGs. **(B)** Venn diagram of GSE31448, GSE45827, and GSE65194. **(C)** protein-protein interaction (PPI) network of DEGs and screening of key genes. The color degree of the node is proportional to the degree of the node. The darker the color, the better the connectivity between genes. **(D)** Using MCODE to cluster in the huge gene (protein) network to construct functional modules, with a total of 14 nodes and 82 edges. The best protein module is selected through the score value of the node by MCODE, and its score value is 12.615. **(E)** Functional analysis of genes, including functional categories or cellular localization. The abscissa is the logarithm of the p-values with a base of 10 and a negative value; the vertical is different enrichment pathways. The higher the -log10 value ranked above, the smaller the p-values and the more significant the enrichment (the darker the color). **(F)** Enrichment analysis of GO and KEGG. Enrichment of key genes in KEGG pathway. The higher the proportion, the more enrichment in the pathway. **(G)** Protein-protein interaction network of DEGs. Selecting more valuable nodes according to degree UnDir and betweenness UnDir. CDK1, NDC80, PLK1, CDC20, AURKB, BUB1 were identified as key genes.

We obtained three sample chips from the GEO database, including 26 healthy breast tissue samples and 164 TNBC tissue samples. The difference analysis of these samples was based on the selection with the adj p < 0.05 and logFC > 2 or logFC < -2 as statistically significant. The volcano map is used to visualize differential genes in order to more intuitively display the expression of differential genes (**Figure 5A**). The drawing of the volcano map is completed by the biological information analysis visualization tool-Sangerbox. After intersecting three groups of differential genes on the Venn diagram, 494 differential genes were obtained (**Figure 5B**).

### Identification of Key Genes

In order to identify disease-related genes, we further screened differential genes. We used STRING database to mine key genes and imported differential genes into the database to form a complex PPI network. The minimum confidence of interaction was set to 0.993, and the screening PPI relational network was preliminarily obtained. Next, the Centiscape plug-in was used for further screening in the Cytoscape software. We selected Betweenness degree > 60, Closeness degree > 0.009, Degree > 15 as the screening criteria for the key genes, and finally obtained four key targets, which are CDK1, NDC80, PLK1, CDC20 (**Figures 5C, G**). We also used MCODE for cluster analysis and obtained an important module from the differential genes. We set Node Score Cutoff at 0.2, K-Core was set to 2 and Max. Depth was set to 100. According to this standard, we sift out a module. This module had a score of 12.615, with a total of 14 nodes and 82 edges (**Figure 5D**). The genes in MCODE cluster analysis contained only four selected genes, which proves that these four genes are valuable in the follow-up analysis.

### Key Gene Expression and Prognostic Analysis

**Figure 6:**
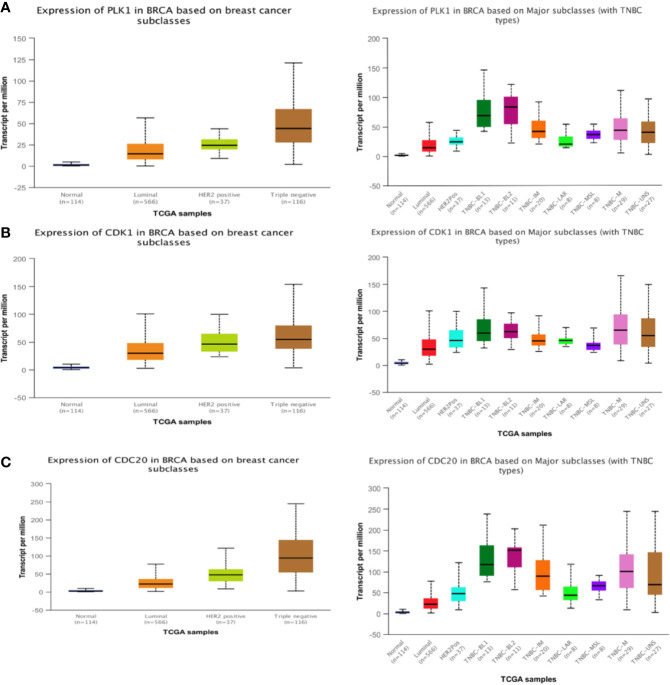
Expression of three hub genes. The expression of PLK1 **(A)**, CDK1 **(B)**, CDC20 **(C)** in triple negative breast cancer is higher than that in other breast cancer tissues.

We put the key genes into the DAVID database and FUNRICH database for GO and KEGG analyses, and found that CDK1, CDC20, and PLK1 are mainly concentrated in the cell cycle pathway, which provided theoretical support and direction for our follow-up research (**Figures 5E, F**). UALCAN was used to study the gene expression of TNBC. This includes expression in normal cells and other breast cancer cells. (**Figures 6A–C**). The key genes we screened out had significantly higher expression in breast cancer than normal tissues, and then higher expression in triple negative breast cancer than other types of breast cancer.

### Molecular Docking

**Figure 7:**
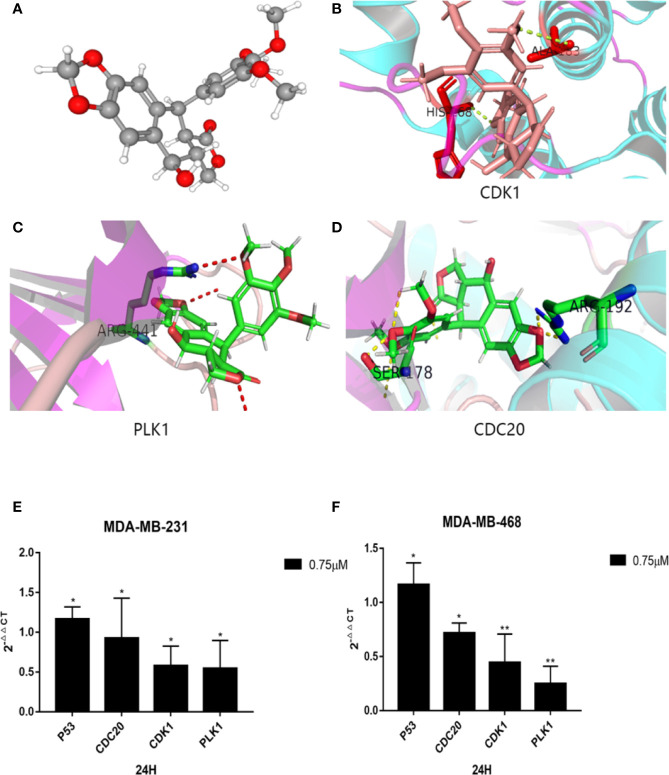
Molecular docking diagram of key genes and mRNA expression. **(A)** 3D model of podophyllotoxin. Schematic diagram of binding sites of **(B)** CDK1, **(C)** PLK1, and **(D)** CDC20. Small molecular proteins are connected to the corresponding sites of the protein through hydrogen bonding. The mRNA expression is regulated by podophyllotoxin in **(E)** MDA-MB-231and **(F)** MDA-MB-468. Cells were treated with 0.75-μM podophyllotoxin, qRT-PCR detection promoted the expression of p53 at the mRNA level and inhibited the expression of CDK1, CDC20, Polo-like kinase1 (PLK1). *p < 0.05, **p < 0.01.

To further investigate the relationship between podophyllotoxin and DEGs, we constructed a molecular docking model to explore the binding site. The molecular docking scores are as follows: PLK1 (PDB: 4o6w, score: 5.45), CDK1 (PDB: 6guh, score: 4.75), and CDC20 (PDB: 4r8q, score: 6.6) and cs score were all greater than five points (**Figures 7A–D**). By exploring the binding position of the largest natural ligand, the binding site of each protein is automatically identified. In the process of preparing proteins, water molecules are removed and hydrogen atoms are added. Docking score, that is, the predicted binding affinity of the target protein. Molecular docking shows that molecular docking studies of compounds are also feasible and will help design new drugs with higher potency based on the rationality of the structure (Gao et al., 2016; Hsin et al., 2016).

### Expression of PLK1, CDC20, CDK1, and P53

According to our analyses, we found that the identified genes are closely related to the cell cycle pathway. Several reports indicated that the upstream and downstream proteins of the cell cycle pathway are regulated by P53 (Zhang et al., 2018; Chen et al., 2019; Wang et al., 2020). Within 24 h after treatment with 0.75 μmol/L podophyllotoxin, we observed the effect of podophyllotoxin on the mRNA level of identified genes in MD-MBA-231 and MD-MBA-468 cells. The levels of cyclins, such as CDC20, CDK1, and PLK1 decreased, while the expression of P53 increased following the treatment. CDC20, CDK1, and PLK1 may be the targets of podophyllotoxin in breast cancer (**Figures 7E, F**). The results were presented as the mean ± standard deviation of triplicate experiments and P<0.05 was considered to indicate a statistically significant difference.

### Analysis of Key Targets

**Figure 8:**
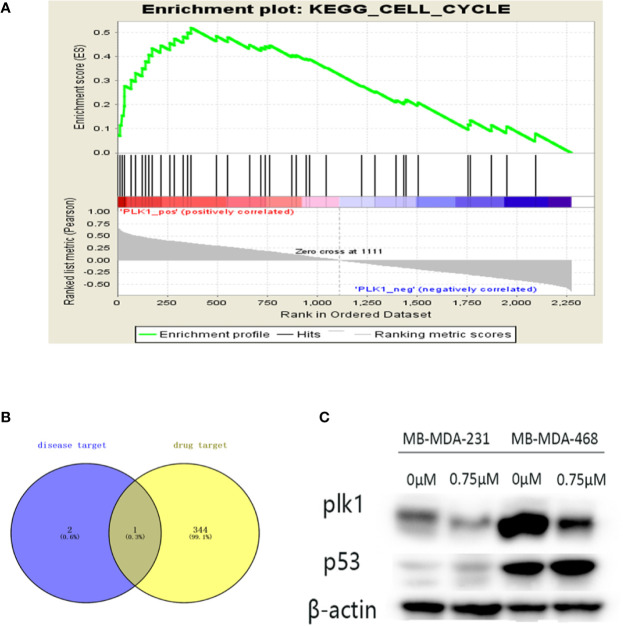
Further screening of key targets. **(A)** Gene Set Enrichment Analysis (GSEA) enrichment of Polo-like kinase1 (PLK1) in cell cycle signaling pathway. NES=2.229, p-value=0, FDR-q-value=0.00213. **(B)** The Venn diagram is the intersection of key disease targets and drug targets. Purple: key target for triple-negative breast cancer (TNBC) disease, yellow: drug target. We end up with a common gene: PLK1. **(C)** The protein expression levels of p53 were assayed by Western blot. The expression of PLK1 protein decreased and the expression of p53 increased with the increase of podophyllotoxin in both cell lines.

By screening the podophyllotoxin targets by BATMAN-TCM and TCMSP, we intersected the disease targets with the drug targets and received the common target PLK1 (**Figure 8B**). Therefore, PLK1 may be a valuable potential target. Thus, we verified it in the GSEA dataset (**Figure 8A**). PLK1 is enriched in the cell cycle pathway. Our further Western blotting results also showed that under the intervention of podophyllotoxin, the protein level of PLK1 decreased with the increase of podophyllotoxin concentration, while the result of P53 level was the opposite (**Figure 8C**). Podophyllotoxin regulates related cyclins by upregulating P53 in breast cancer. Combined with the application of network pharmacology, we suggest that PLK1 may be a more valuable target as a drug to combat this disease.

## Discussion

We found that podophyllotoxin can inhibit the proliferation, migration and invasion of breast cancer. Podophyllotoxin can induce apoptosis and block cell cycle. In order to further explore the action mechanism of podophyllotoxin, we used GEO database, protein interaction network, and network pharmacology to predict the key target genes. KEGG analysis showed that three key genes are mainly enriched in the cell cycle pathway. Using Kaplan-Meier mapping instrument to access the gene chip database, we found that CDK1, PLK1, and CDC20 are associated with poor prognosis of breast cancer, which suggests that these three genes may be valuable genes. With the help of molecular docking, we can visualize the binding sites of ligands and receptors, and can further understand their binding sites, hydrophilicity and hydrophobicity, and affinity and sparse charge. At the same time, the binding site map of protein and podophyllotoxin is to verify the results of data analysis. We verified that podophyllotoxin downregulates the expression of three target genes at the mRNA level. We intersected the disease and drug targets then obtained the common target PLK1. PLK1 may be a more valuable potential target. Finally, the Western blot analysis confirmed the effect of podophyllotoxin on breast cancer by interfering with PLK1 on the protein level.

TNBC is characterized by the lack of ER, PR, and HER2 expression (Amos et al., 2012). In the current treatment process, hormone therapy and targeted therapy have no ideal therapeutic effect on TNBC (Criscitiello et al., 2012). TNBC is a significantly heterogeneous disease not only on the molecular level but also from the pathological and clinical view (Metzger-Filho et al., 2012). TNBC shows higher aneuploidy or proliferation, in addition to more invasive biological characteristics (Malorni et al., 2012). Although cytotoxic chemotherapy, neoadjuvant therapy of vascular endothelial growth factor (VEGF) inhibitors, EGFR inhibitors, and tubulin inhibitors may play a specific role in the treatment of TNBC, their action mechanisms and benefits in TNBC are not well-understood, requiring further exploration (von Minckwitz and Martin, 2012). Therefore, the study of therapeutic drugs that can interfere with the key targets of TNBC remains a main focus of the research. Podophyllotoxin and its derivatives have extensive effects on the biological system, including cell metaphase arrest, cell differentiation, or cell membrane transport (Sackett, 1993). Podophyllotoxin is also an antitubulin drug, which can prevent cell division (Li et al., 2019). Podophyllotoxin is considered to be a potential anticancer drug. By preventing the polymerization of tubulin, podophyllotoxin inhibits the cell division phase and induces the cell cycle to stay in mitosis (Loike and Horwitz, 1976a; Ardalani et al., 2017). In addition, podophyllotoxin and podophyllotoxin derivatives have different effects on microtubule assembly and DNA fragmentation. Podophyllotoxin does not cause cell DNA fragmentation and inhibits microtubule assembly *in vitro* (Loike and Horwitz, 1976b). Related research shows that the semi-derivatives of podophyllotoxin combined with other drugs are widely used in the clinical treatment of small cell lung cancer and lymphoma (Najar and Johri, 2014). At present, there are few studies on breast cancer with podophyllotoxin. However, on the basis of previous studies, we found that there is association between cell cycle division in TNBC and the mechanism of podophyllotoxin. Thus, we conducted an exploratory study of podophyllotoxin on TNBC.

Podophyllotoxin can inhibit the proliferation of breast cancer and reduce the metastatic and invasive ability of breast cancer cells. Nevertheless, little is known about the effect of podophyllotoxin on breast cancer metastasis, which also provides a new research direction for podophyllotoxin action on TNBC. In addition, we found that podophyllotoxin can mainly block the cell cycle in the G2/M phase and induce cell apoptosis. This is consistent with the results confirmed by previous researchers that podophyllotoxin and its analogs can block lung cancer cells in the G2/M phase and induce tumor apoptosis (Antunez-Mojica et al., 2016). Podophyllotoxin acts on TNBC, resulting in DNA damage, cell cycle checkpoints, and an increase in the number of cells in the G2/M phase. Cell death experiments showed that apoptosis increased significantly after the addition of drugs. Cell cycle arrest and apoptosis induction is one of the characteristics of delaying tumor progression. Targeting the deregulation of cell cycle and checkpoint machinery in cancer offers the possibility of cancer treatment (Aarts et al., 2013).

We analyzed the differences in gene expression of GSE31448, GSE45827 and GSE65194 datasets in the GEO database. From that, 566 differential genes were identified, and then three key target genes were obtained by protein interaction network, cluster analysis, and molecular docking, namely CDK1, PLK1, and CDC20. Through the UALCAN database, we found that CDK1, PLK1 and CDC20 are highly expressed in many tumors. CDK1 is a major member of the (CDK) family of cyclin-dependent protein kinases (Brown et al., 2015). CDK1 has potential applications in many fields. Some studies have shown that the cycle of apoptosis induced by CDK1 can be inhibited by increasing the expression level of p21 and P53 (Gao et al., 2019). As a tumor-promoting factor, CDC20 plays an important role in regulating the cycle. Upstream regulatory factors of CDC20, including P53, Emi1, and USP44, can block its carcinogenic effect. Inhibition of CDC20 may be a target of tumor therapy (Wang et al., 2015).

Through the integration of network pharmacology-related databases and breast cancer target genes, we found that PLK1 is not only the target gene of breast cancer but also the target gene of podophyllotoxin. Moreover, GSEA also revealed that PLK1 plays a key regulatory role in the cell cycle. PLK1 was first found in Drosophila polo gene mutations and plays an important conservative role in mitosis (Archambault and Glover, 2009). Polo-like kinase1 (PLK1) is a typical member of the serine/threonine kinase Polo-like family (Combes et al., 2017). PLK1 is essential for cell cycle progression throughout the cell cycle (Rape, 2007). PLK1 has multiple roles in the cell cycle, especially in the mitotic G2/M transition, bipolar spindle formation, chromosome separation, and cytokinesis. Not only that, PLK1 regulates spindle assembly, chromosome segregation, promotes DNA replication and participates in meiosis (Barr et al., 2004). It plays a role by activating CDK1 and cyclin B when cells enter the M phase(Archambault and Glover, 2009). More importantly, PLK1 plays a more critical role in tumor development. The imbalance between PLK1 and tumor suppressor protein may accelerate the progression of the tumor (Eckerdt et al., 2005). Increased PLK1 DNA breakage, G2-M phase arrest, increased apoptosis, cell viability and tumorigenicity are suppressed, these phenomena will occur when PLK1 is inhibited in TNBC (Maire et al., 2012; Garcia et al., 2020). In addition, PLK1 inhibitors also seem to have advantages over mitotic inhibitors (such as taxane or vinblastine) because they do not induce neurotoxicity (Hu et al., 2012). However, when PLK1 inhibitors are combined with taxane and other chemotherapeutic drugs, there will be a synergistic effect. Combined use of PLK1 inhibitors and antimicrotubule drugs such as taxane is important for patients with TNBC resistant to chemotherapy (Giordano et al., 2019). And through experiments we found that podophyllotoxin can inhibit the expression of PLK1. Patients with TNBC can benefit from drugs involving PLK1 inhibitors.

Previous studies have confirmed that PLK1 interacts with tumor suppressor gene P53 in mammalian cells (Ando et al., 2004). PLK1 is considered as an attractive target for tumor intervention therapy. Inhibition of PLK1 activity can block the mitotic phosphorylation of CdC25 and the activation of CDK1, thus increasing the expression of P53 and selectively kill tumor cells (Conn et al., 2000). These three key genes are enriched in the cell cycle signaling pathway and are further regulated by the P53 pathway. P53 is closely related to cyclin. P53 is a common mutant gene in human tumors (Levine, 1997). The main function of the P53 tumor suppressor gene is to regulate the transcriptional control of target genes in many cell processes, including cell cycle and apoptosis. Besides, P53 is activated during stress signals, DNA damage, and oncogene activation, which leads to growth inhibition by inducing cell cycle arrest or cell death (Fischer, 2017). Podophyllotoxin affects cell cyclins by regulating P53. In addition, previous literature studies also support the regulation of P53 on PLK1, such as plk1 and P53 are related to many cyclin content (Sanhaji et al., 2013). PLK1 is likely to be the target of P53, and P53 can mediate these effects by directly inhibiting the expression of PLK1 (McKenzie et al., 2010). The results of qRT-PCR showed that the expression of P53 increased at the mRNA level, while the mRNA levels of CDK1, PLK1, and CDC20 were decreased. Podophyllotoxin can reduce the expression of related cyclins by upregulating the mRNA level of P53. CDK1, CDC20, and PLK1 enable the effective mechanisms of podophyllotoxin cell cycle arrest in the G/2M phase. Among them, PLK1 may be a more exploratory target. It can be used not only as prognostic indicator, but also as a starting point for tumor treatment.

Our study reflects that CDK1, PLK1, and CDC20 are potential therapeutic targets of podophyllotoxin in breast cancer; among them, PLK1 is more worthy of further exploration. In our study, we provide a new insight for podophyllotoxin activity on the metastasis of TNBC. The combination of natural compounds and disease targets in exploring the mechanism of the cell cycle can provide a new idea for follow-up research. Bioanalysis technology on the molecular level provides a feasible strategy and method to thoroughly explain the mechanism of natural medicine components interfering with diseases. It generates an idea for exploring new targets of podophyllotoxin in the treatment of breast cancer, and it also provides a new angle for the design and synthesis of podophyllotoxin.

## Conclusion

In this study, we carried out cell experiments on foot leaf toxin and confirmed that foot leaf toxin has a certain effect on breast cancer. Podophyllotoxin can inhibit cell proliferation, migration and invasion. In addition, our research innovatively proposed that podophyllotoxin effectively regulates the TNBC cell cycle and promotes cell apoptosis. Through the comprehensive analysis of the databases, we found that the expression of CDC20, CDK1, and PLK1 are increased in breast cancer. Podophyllotoxin can inhibit the expression of CDC20, PLK1, and CDK1 and increase the expression of P53. After further screening, we focused on the analysis of PLK1, specifically, it may be through the up-regulation of key targets P53 and down-regulation of PLK1, thereby inhibiting the further development of tumors. This study initiates a new direction for exploring the effect of podophyllotoxin on breast cancer and provides theoretical support for further exploration of podophyllotoxin.

## Data Availability Statement

Publicly available datasets were analyzed in this study. This data can be found here: GSE65194, GSE45827, GSE31448. The data can be found in https://www.ncbi.nlm.nih.gov/geo/.

## Author Contributions

WZ CL, and CS conceived of the presented idea. Data mining and gene analysis were carried out by JL, YY, and RL. WZ, JZ, and FF performed relevant experimental operations. WZ, CL, and CS wrote the manuscript. CL, JL,WZ contributed to the manuscript revision. All authors contributed to the article and approved the submitted version.

## Funding

This work is supported by the grants from National Natural Science Foundation of China (81673799) (81973677) and National Natural Science Foundation of China Youth Fund (81703915).

## Conflict of Interest

The authors declare that the research was conducted in the absence of any commercial or financial relationships that could be construed as a potential conflict of interest.

## Supplementary Material

Supplementary Image 1 **(A)** Pathway analysis of key genes. **(B) (C)** Western gray analysis of key genes.

Supplementary Image 2 Targets for genes and drugs.

Supplementary Image 3 Verification of TCGA-related genes.

Supplementary Image 4 Clinical information and chip status of GSE45827, GSE65194, GSE31448 chips.
