# Supplementary figures and images for: Target Analysis and Mechanism of Podophyllotoxin in the Treatment of Triple-Negative Breast Cancer
Source: Front Pharmacol. 2020 Aug 7;11:1211. doi: 10.3389/fphar.2020.01211 (PMC7427588; doi:10.3389/fphar.2020.01211)

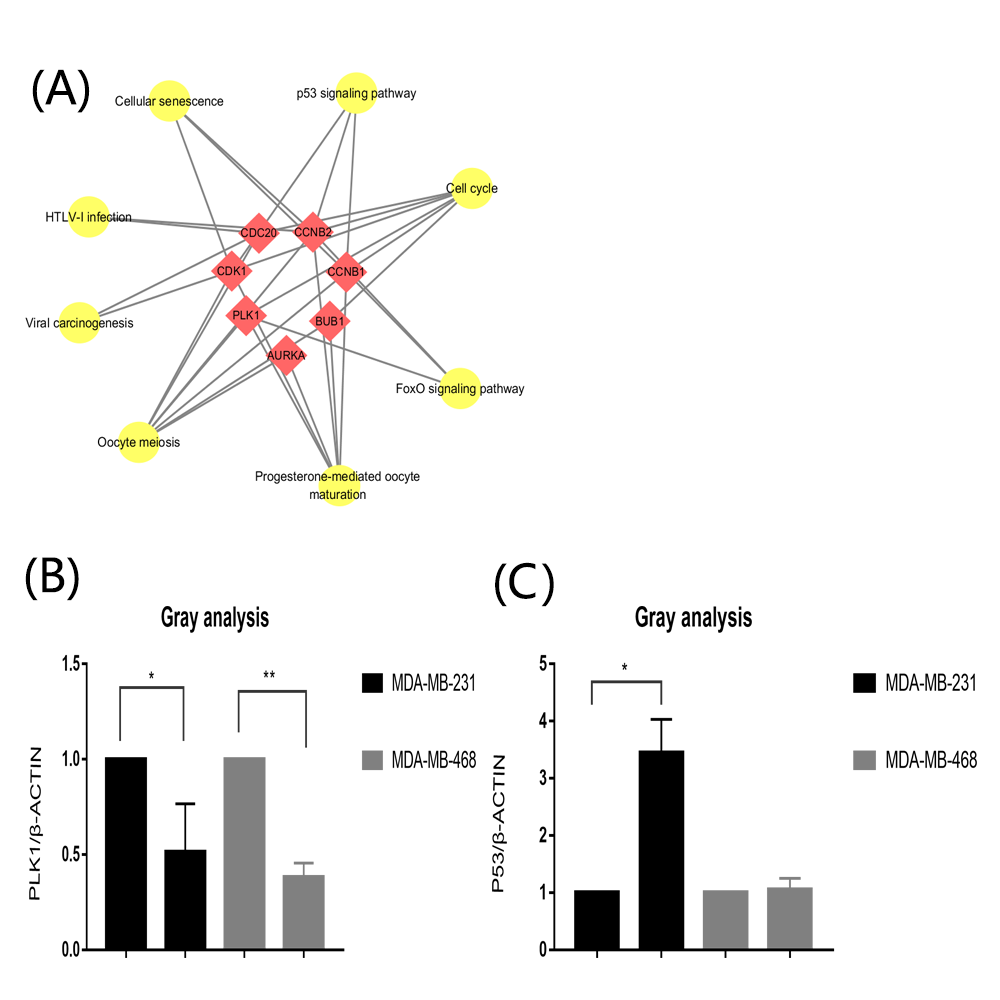

Supplement: Supplementary Image 1 — (A) Pathway analysis of key genes. (B) (C) Western gray analysis of key genes. [file Image_1.tif]

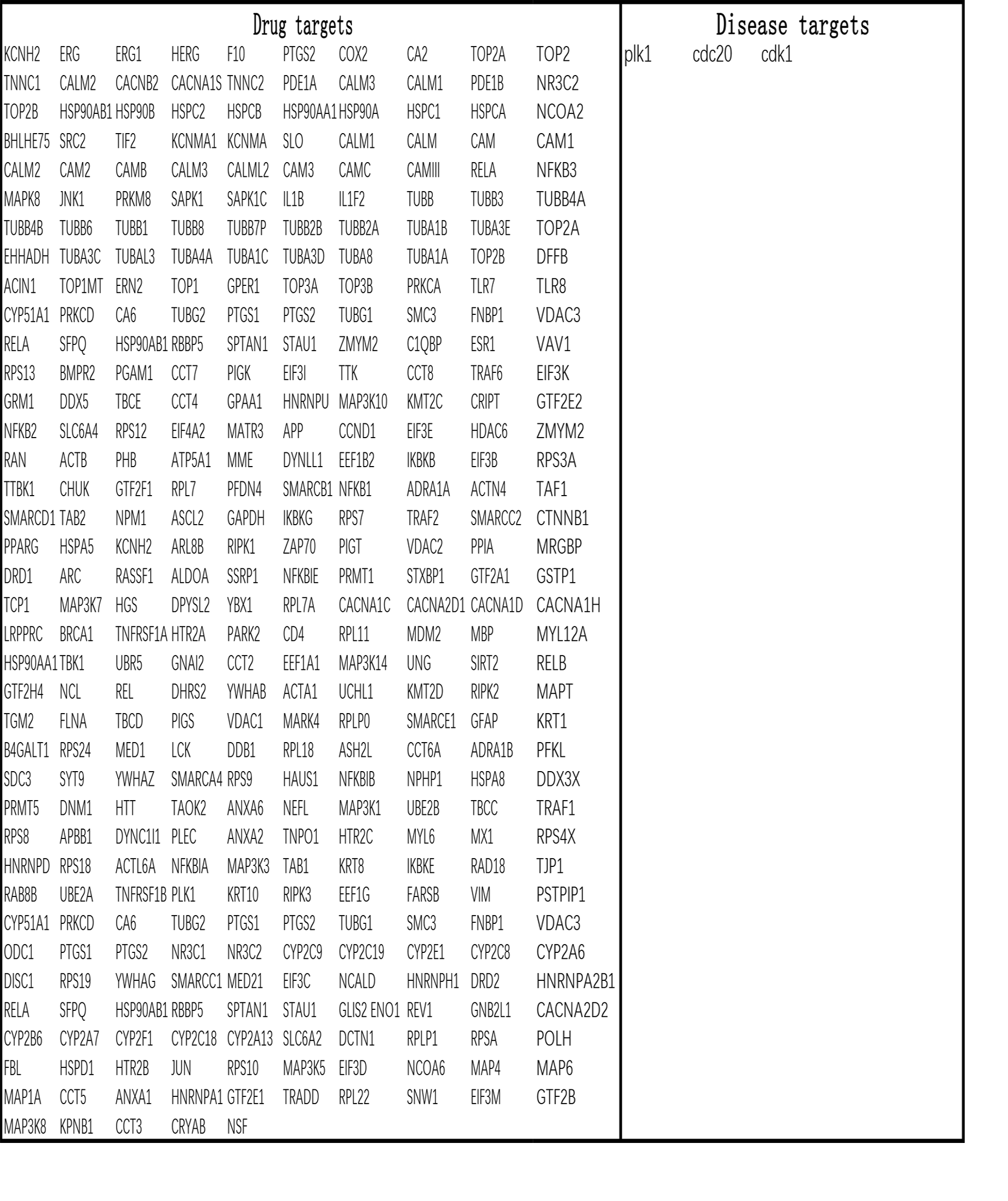

Supplement: Supplementary Image 2 — Targets for genes and drugs. [file Image_2.tif]

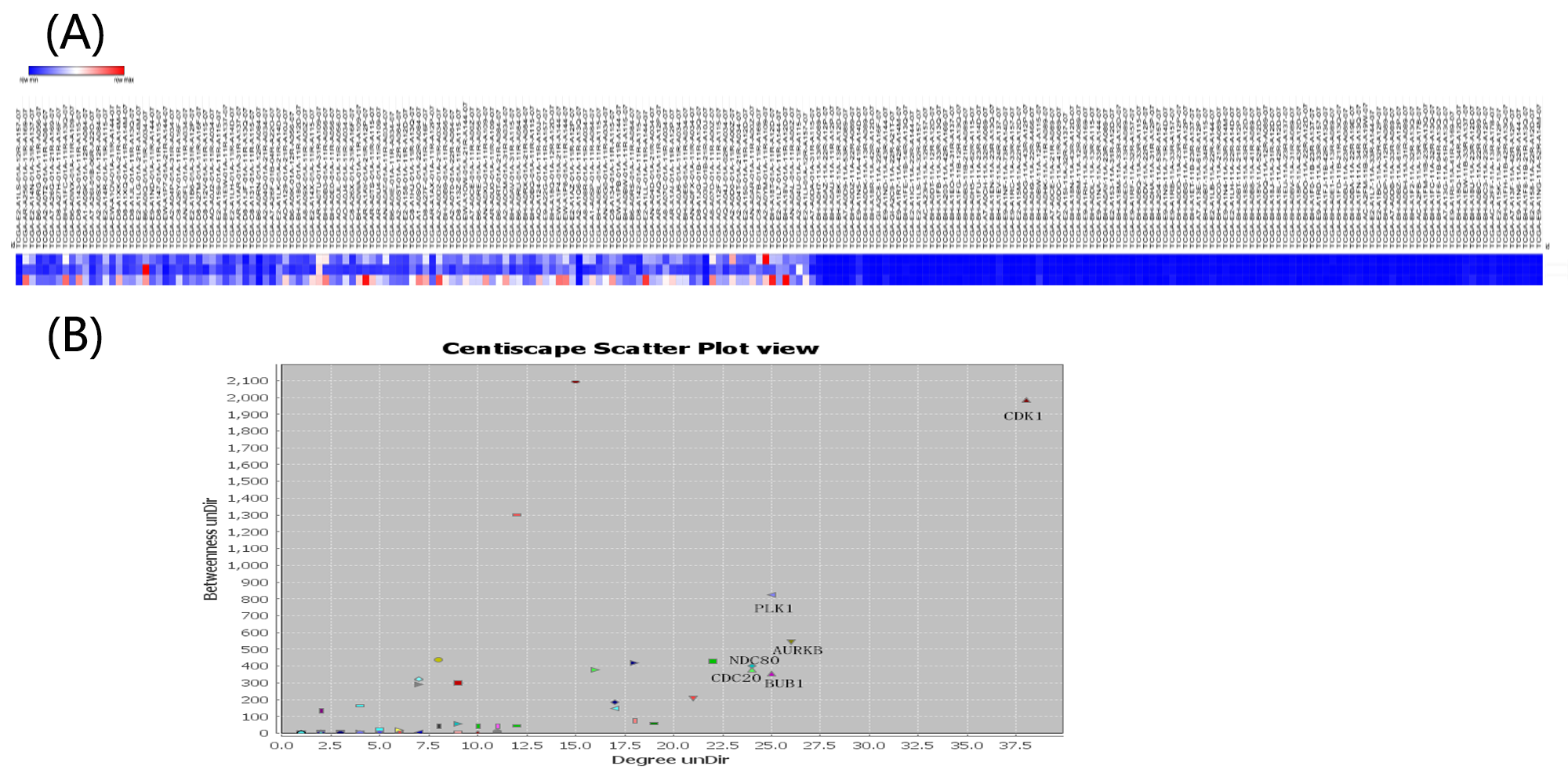

Supplement: Supplementary Image 3 — Verification of TCGA-related genes. [file Image_3.tif]

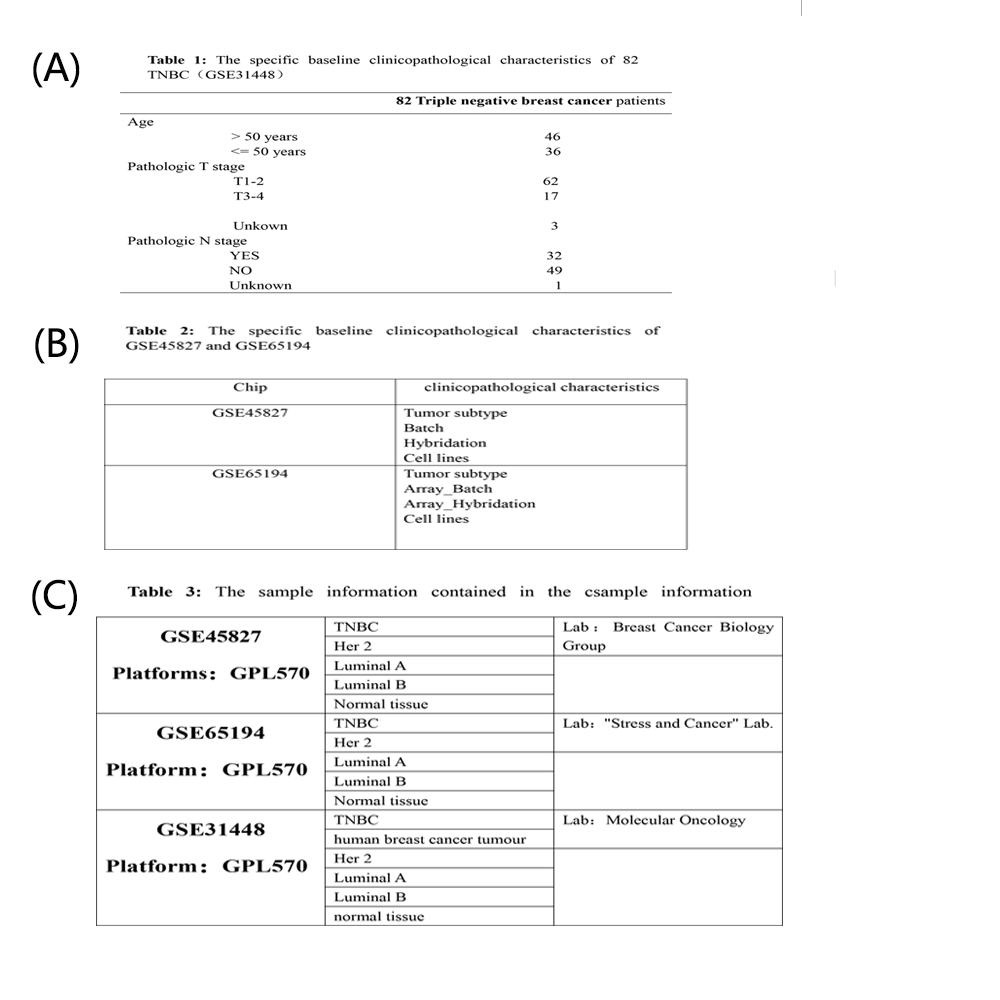

Supplement: Supplementary Image 4 — Clinical information and chip status of GSE45827, GSE65194, GSE31448 chips. [file Image_4.tif]
